# Oiling accelerates loss of salt marshes, southeastern Louisiana

**DOI:** 10.1371/journal.pone.0181197

**Published:** 2017-08-02

**Authors:** Michael Beland, Trent W. Biggs, Dar A. Roberts, Seth H. Peterson, Raymond F. Kokaly, Sarai Piazza

**Affiliations:** 1 Department of Geography, San Diego State University, San Diego, California, United States of America; 2 Department of Geography, University of California Santa Barbara, Santa Barbara, California, United States of America; 3 United States Geological Survey, Denver, Colorado, United States of America; 4 United States Geological Survey, Wetland and Aquatic Research Center, Baton Rouge, Louisiana, United States of America; Centro de Investigacion Cientifica y de Educacion Superior de Ensenada Division de Fisica Aplicada, MEXICO

## Abstract

The 2010 BP Deepwater Horizon (DWH) oil spill damaged thousands of km^2^ of intertidal marsh along shorelines that had been experiencing elevated rates of erosion for decades. Yet, the contribution of marsh oiling to landscape-scale degradation and subsequent land loss has been difficult to quantify. Here, we applied advanced remote sensing techniques to map changes in marsh land cover and open water before and after oiling. We segmented the marsh shorelines into non-oiled and oiled reaches and calculated the land loss rates for each 10% increase in oil cover (e.g. 0% to >70%), to determine if land loss rates for each reach oiling category were significantly different before and after oiling. Finally, we calculated background land-loss rates to separate natural and oil-related erosion and land loss. Oiling caused significant increases in land losses, particularly along reaches of heavy oiling (>20% oil cover). For reaches with ≥20% oiling, land loss rates increased abruptly during the 2010–2013 period, and the loss rates during this period are significantly different from both the pre-oiling (p < 0.0001) and 2013–2016 post-oiling periods (p < 0.0001). The pre-oiling and 2013–2016 post-oiling periods exhibit no significant differences in land loss rates across oiled and non-oiled reaches (p = 0.557). We conclude that oiling increased land loss by more than 50%, but that land loss rates returned to background levels within 3–6 years after oiling, suggesting that oiling results in a large but temporary increase in land loss rates along the shoreline.

## Introduction

Coastal wetlands provide a myriad of important ecosystem services, including flood mitigation, pollution removal, carbon sequestration, wildlife habitat and recreational opportunities, but they are threatened by an array of human activities, both directly by dredging, channelization and construction, and indirectly by sea level rise and reduced sediment input. Intertidal ecosystems, particularly salt marshes, are resilient to physical disturbances, which has been attributed to their high productivity [1,2,3] and their physiological traits for coping with stressful environmental conditions [4,5,6,7,8].

For nearly two centuries, human activities in the northern Gulf of Mexico have altered natural hydrologic regimes and changed the magnitude of system perturbations beyond salt marsh resilience thresholds [9,10], resulting in accelerated rates of wetland loss (> 250 km^2^ yr^-1^) [11]. Since the 1970's, land loss has been a major topic of concern with broad management implications for the region, particularly for coastal Louisiana [12,13,14,15,16]. Louisiana alone lost an estimated 4800 km^2^ of intertidal wetland area from 1932–2010 (∽62 km^2^ yr^-1^) [17]. A combination of natural (e.g. subsidence, sea-level-rise, abandoned river delta decay, wave energy and storm events) and anthropogenic (e.g. levees, impoundments, canal dredging and subsequent channel erosion) forces have contributed to the alarming rates of wetland loss, which has been popularly expressed in media outlets as "a football field per hour" [17].

Barataria Bay, a rapidly eroding abandoned delta where aggradation is no longer keeping pace with the effects of eustasy and subsidence [18], perhaps best illustrates the challenge of managing land loss [16,19], as it has been losing 15.1 km^2^ of wetland area per year since 1932 [17,20]. Yet, these land losses in the Barataria Basin have not increased monotonically over the past century [17,21]. Episodic disturbances, like oil spills and hurricanes, can accelerate land loss, particularly along marsh edges, in areas already experiencing marsh degradation or loss [22,23,24,25,26].

The largest oil spill in U.S. history occurred in the Gulf of Mexico on April 20, 2010, when an explosion on the Deepwater Horizon (DWH) offshore drilling unit released 780,000 m^3^ of crude oil into the Gulf before being capped on July 15 [27]. Oil washed onto approximately 796 km of shoreline comprised of intertidal marshes, disproportionally impacting salt marshes of Louisiana [28]. Oiling was concentrated along the marsh shoreline edge [25,29,30], causing plant stress, mortality, and reductions in above- and belowground biomass [26,31]. Exposure of marsh macrophytes to oil can lead to reduced function (i.e. transpiration and photosynthesis) followed by recovery through new shoot regeneration [32,33], or plant mortality and reduced biomass production, resulting in destabilization of the root-soil matrix [7,25]. Soil strength and sediment accretion are directly related to belowground biomass as roots and rhizomes create a binding matrix for sediment accumulation [21,34,35]. Reductions in belowground biomass caused by oiling and subsequent remediation efforts increases the vulnerability of shorelines to both episodic (i.e. storm surge) and chronic (i.e. subsidence, sea-level rise) erosional forces [25,26,36,37].

Barataria Bay was among the areas most heavily impacted by oil following the DWH spill [28]. The threat of accelerated erosion is of particular concern for the rapidly deteriorating marsh platforms of the lower Barataria Basin [25,26,31,36]. Land loss in the lower basin over the last century has been caused by a combination of natural and anthropogenic erosional forces, including reduced sediment deposition from the Mississippi River, compaction and subsidence of underlying deltaic deposits, flood control practices and canal dredging [16,23,38]. Oiling from the DWH spill in Barataria Bay was concentrated within the first 15 m from the marsh edge (maximum of 19m) [29,30], with only 1% reaching beyond 15m [39]. Oiling accelerated shoreline erosion, contributing to erosion rates at oiled sites that were more than double that of reference (non-oiled) sites a year after exposure [25,36]. However, existing studies were conducted over relative small areas (60 m of shoreline in Silliman et al. [25]; 300 m of shoreline in McClenachan et al. [26]; ∽630 m of shoreline in Zengel et al. [36]). Extrapolating results from these small study areas to regional scales can be problematic, due to the variability in shoreline orientation and exposure to wave action, degree of oiling, and variable responses of aboveground and belowground biomass to oiling [26,31,40]. Consequently, the magnitude of marsh shoreline retreat and land loss attributed to oiling is difficult to quantify over regional scales by extrapolating from specific study reaches.

Three recent studies used remote sensing techniques to examine the impacts of oiling on salt marshes of Barataria Bay on a landscape-scale [41,42,43]. Beland et al. [41] found that only *Spartina alterniflora* dominated marshes were extensively degraded and that vegetation classes converted to an open water class along oiled shorelines at more than double the rate of non-oiled shorelines from 2010–2012. In comparing pre-oiling (2009–2010) shoreline recession rates, Rangoonwala et al. [42] documented a fourfold and threefold increase in shorelines experiencing >4 m recession for the first and second years after oiling. Turner et al. [43] assessed shoreline loss by measuring the change in width (east-west) and length (north-south) of 46 marsh islands in Barataria Bay, and reported erosion rates of oiled islands were 3 times that of non-oiled islands for the first 2.5 years after oiling. To date, however, a bay-wide and reach-scale assessment of wetland loss attributable to oiling has yet to be conducted. Further, previous studies have not accounted for variability in background erosion rates for oiled shorelines, or determined if land loss rates remained above pre-oiling rates or returned to background levels beyond the first 2.5 years.

In this study, our objectives were to: a) map changes in land loss along the shoreline in a bay affected by the DWH oil spill for three time periods: before, 3 years after, and 6 years after the spill, b) determine if rates of land loss were significantly different before and 3 and 6 years after the spill, and c) quantify the impact of oiling on reach-scale and bay-wide loss rates, controlling for temporal variability in natural background erosion rates. Land loss rates per unit shoreline (m^2^ m^-1^ yr^-1^) were calculated to standardize the loss rates for varying shoreline lengths, and to provide results that can be easily compared with future assessments of marsh loss along the shoreline in the Louisiana Coastal Zone. The rationale for examining the land loss rates at three year time intervals derived from the temporal response patterns documented in prior research [42,43]. Additionally, the time intervals (i.e. length of time between image acquisition dates) were constrained by the availability of high resolution satellite and airborne datasets capturing Barataria Bay.

## Materials and methods

We used a combination of remote sensing and GIS techniques and simple statistical algorithms to map shoreline change. Marsh shorelines were segmented into non-oiled (i.e. reference) and oiled reaches, and land loss rates were calculated to determine if loss rates were significantly different for oiled and non-oiled reaches, and for pre- (3 years before oiling) and post-oiling (0–3 years, 3–6 years) time periods. Last, we calculated background land loss rates for oiled reaches, using a combination of oiled (pre-spill) and non-oiled (pre- and post-spill) shoreline loss rates, to estimate the magnitude of oil-related land losses that are not attributable to temporal variability in background loss rates.

### Study area description

**Fig 1:**
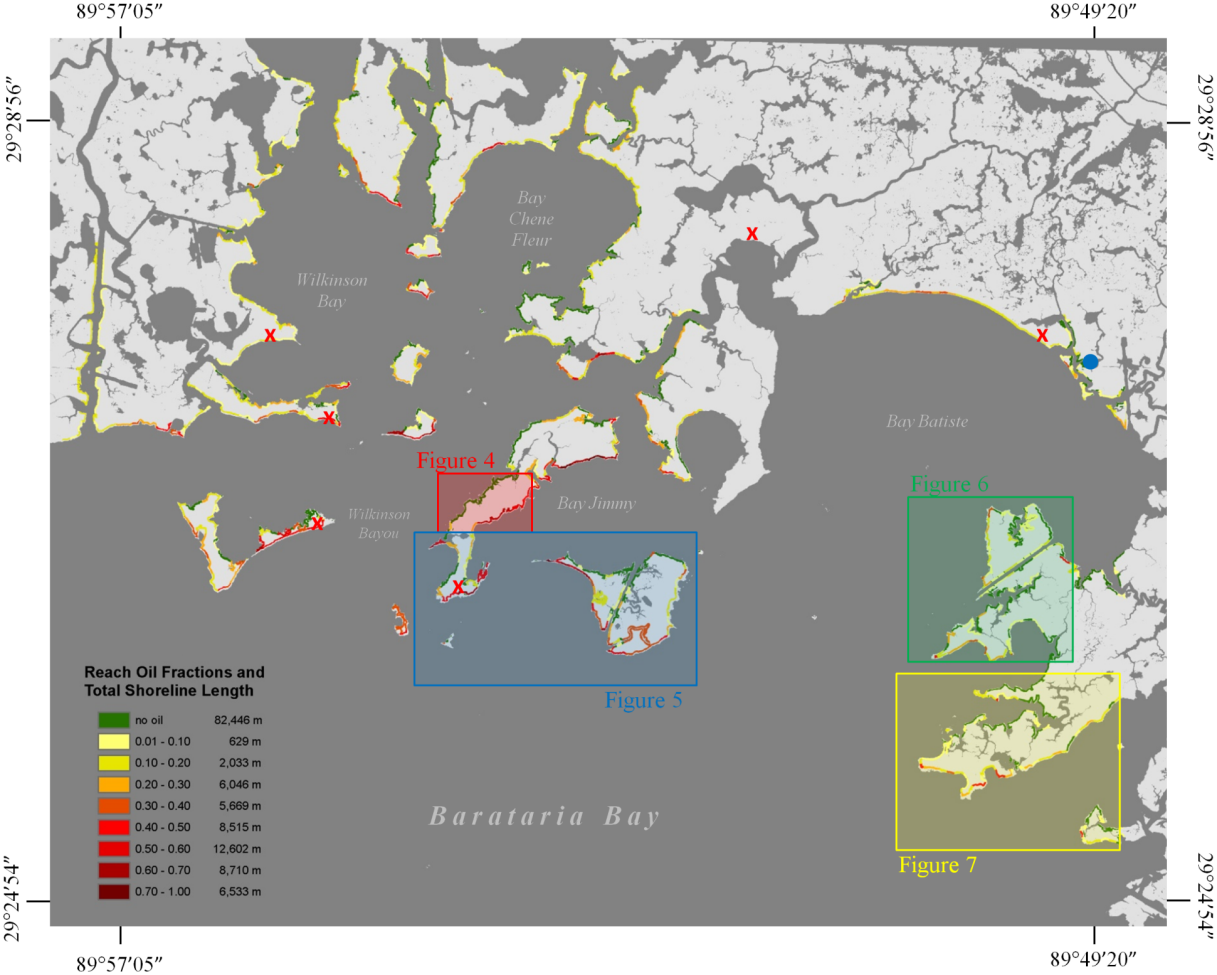
Upper Barataria Bay study area. Shows shoreline reach oil fractions, NDVI validation locations (red x’s), water level measurement site (blue circle).

The study area covers approximately 197 km^2^ in northern Barataria Bay, Louisiana (29.43°N, 89.88°W), and consists of 41 km^2^ of marsh area and 133 km of marsh shoreline (excluding interior channel and canal banks) (Fig 1). Barataria Bay is an interdistributary bay, formed between the active Plaquemines delta lobe and Lafourche headland, which is experiencing some of the highest relative sea level rise rates in the continental United States (0.94 cm/yr from 1947–2006) [19]. Salt marshes of Barataria Bay are fractions of a meter from sea level and are being impacted by sea level rise [44], and are highly vulnerable to natural and anthropogenic disturbances [16,23,45].

Soils in the lower Barataria Basin form on sediment and are tidally redistributed in the lower basin [46]. Soils (Timbalier, Lafitte, Bellpass, Clovelly, Scatlake series) are very poorly drained and consists of a moderate to thick layer (30–310 cm) of muck and fibrous peat (20% organic content) over clayey (coarse silt) alluvium with 0–0.2% slopes [47]. The lower Barataria Basin is a microtidal environment with a diurnal spring tidal range less than 0.6 m [46]. Diurnal tides and wind-driven winter storms account for frequent water exchanges between the lower Barataria Basin marshes and the Gulf of Mexico, while tropical storms account for infrequent, yet pronounced flooding of the marsh platform with saline water [48].

Salt marshes of Barataria Bay are vegetated by dense monotypic stands of polyhaline and mesohaline macrophytes, with *Spartina alterniflora* and *Juncus roemerianus* commonly comprising more than 80% of the vegetation cover. *Distichlis spicata*, *Spartina patens*, *Phragmites australis*, *Schoenoplectus americanus* and *Schoenoplectus robustus* are also common [31,41,49]. Non-inundated bare soil (e.g. mudflats, salt pannes, unvegetated marsh edges) cover accounts for < 2% of the total marsh area [41].

### Oil fraction cover maps

The oil maps used here were generated using MESMA applied to Airborne Visible/Infrared Imaging Spectrometer (AVIRIS) imagery and published in Peterson et al. [39]. AVIRIS datasets were radiometrically calibrated, converted to apparent surface reflectance using Atmospheric Correction Now (ACORN 6.0, ImSpec LLC, Seattle), and ground-reflectance spectra from a calibration site (airport tarmac) were used to remove residual atmospheric features [39]. Peterson et al. [39] used iterative endmember selection (IES) [50] to produce a spectral library of green vegetation, non-photosynthetic vegetation, soil and oiled marsh endmembers. Stable Zone Unmixing (SZU) [51], the InStability Index (ISI) [52] and synthetic mixture modeling were used to identify an optimal subset of nine bands for discriminating endmembers, particularly bands that effectively separated spectrally similar oiled marsh and non-photosynthetic vegetation. Finally, two, three and four endmember models were run on each image, followed by an automated extraction process in which endmember combinations with the lowest RMSE and least complexity (fewest endmembers) were selected for each pixel and merged into a multiple endmember fractional cover dataset. The models were run on images from July 31, August 15, September 14, October 4, 2010 and May 4, 2011 to capture the movement of oil around Barataria Bay [39]. Accuracies for the image dates ranged from 87.5% to 93.3% with zero false positive detections [39]. Here, we created marsh oiling zones of 0-21m from the shoreline edge, and extracted the maximum oil fraction (per 3.5 m pixel) over a multi-temporal data set of oil maps (i.e. July 31, August 15, September 14, October 4, 2010 and May 4, 2011). Overall, the oil maps used here were consistent with the Shoreline Cleanup Assessment Technique (SCAT) maps used in previous studies [28,42], however, some discrepancies in oil coverage along shorelines were apparent. These variations were likely due to differing methodologies, reach extents and oil surface cover categories.

### Mapping shoreline change: Remote sensing techniques

High resolution (0.30–0.64 m) orthorecitifed image datasets were acquired from DigitalGlobe (https://www.digitalglobe.com) and Aerometric Inc. (http://gis.aerometric.net/dirlists.htm) for the four dates used in this study (S1 Table). The DigitalGlobe products were captured on the QuickBird-2 and WorldView-2 & 3 instruments (panchromatic and multispectral) at ground sample distances (GSD) ranging from 0.31m to 0.64m (Table 1). Aerometric Inc. four band (blue, green, red, near infrared) stereoscopic photographs have a GSD of 0.30m (RMSE < 1.2 m). A relative image-to-image accuracy of 0.77 m (RMSE) was achieved across all image dates.

**Table 1:** Annual land loss rates by reach oiling category and time period. Rows (oiling categories) and columns (time periods) also include background annual loss estimates and resulting p-values for the reach-level pair-wise T-tests for Period 1 (2006–2010), Period 2 (2010–2013) and Period 3 (2013–2016) and for each oil category.

oil category	shoreline length (m)	Annual land loss rate 2006–2010 (m^2^ yr^-1^)	Annual land loss rate 2010–2013 (m^2^ yr^-1^)	2010–2013 *background*	Annual land loss rate 2013–2016 (m^2^ yr^-1^)	2013–2016 *background*	Periods 1 & 2	Periods 2 & 3	Periods 1 & 3
no oiling	82,446	43,348	58,762	58,762	51,904	51,904	0.129	0.249	0.581
>0–10	629	29	402	39	132	34	0.146	0.302	0.095
10–20	2,033	385	1,070	522	1,074	461	0.253	0.910	0.143
20–30	6,046	2,254	7,351	3,056	2,378	2,699	***0*.*001***	***0*.*002***	0.849
30–40	5,669	1,923	9,481	2,607	2,313	2,302	***<0*.*001***	***0*.*002***	0.484
40–50	8,515	6,973	20,461	9,453	5,550	8,350	***0*.*006***	***<0*.*001***	0.368
50–60	12,602	9,771	29,840	13,245	9,701	11,699	***<0*.*001***	***<0*.*001***	0.357
60–70	8,710	6,022	16,748	8,163	7,626	7,211	***<0*.*001***	***<0*.*001***	0.616
70+	6,533	4,087	9,792	5,540	4,749	4,894	***0*.*023***	***0*.*046***	0.248
Total:	133,183	74,792	153,909	101,387	85,427	89,553	***<0*.*001***	***<0*.*001***	0.56

We generated binary classification maps of marsh cover and open water for each image (S2 Fig). Marsh vegetation cover and open water are easily distinguishable in bands 4 (NIR) and 3 (red), so we utilized the Normalized Difference Vegetation Index (NDVI), and a binary threshold of -0.03 to create marsh land and open water cover classification maps. A -0.03 threshold was used to include mudflats in the marsh land class. Maps were assessed using field observations made contemporaneous with image acquisition dates (n = 289) from six field sites located along the marsh edge (Fig 1). Land and water were classified accurately for 97% of the observations. Mudflats and lakes located within the marsh interior that had no connectivity with large channels and bays were removed by converting the raster of water pixels into a polygon. This process was followed for all four image dates. Post-classification change detection analysis was performed to determine if marsh area was retained from the previous imaging date, or if a conversion from marsh to open water (i.e. land loss) had occurred.

Image acquisition time could affect the amount of water mapped due to tides, therefore, image data captured at or below mean low water (MLW) are ideal and acquisition at 1–2 feet (0.31–0.62 m) above MLW is acceptable for the northern Gulf of Mexico [53,54]. Here, acquisition times for the data were at 17:10 (2006), 17:00 (2010), 21:25–21:31 (2013) and 16:44 UTC (2016), corresponding to tidal heights of 0.007, -0.031, 0.140 and 0.185 m from MLW (S1 Table). The tidal range (0.22 m) over all image acquisition periods is relatively small, and the maximum tidal height of 0.185 m above MLW (2016) is well within the preferred tidal range (< 0.31 m) stated previously [53,54]. In addition, the ratio of erosion in oiled and non-oiled reaches should be insensitive to tidal effects because the background rate for non-oiled reaches is determined from the same image pair as the oiled reaches (see *Land loss analysis* section).

### Land loss analysis

Image change analysis often uses pixel-wise comparisons over time. For analysis of marsh land loss along shorelines, both total area loss and the distance of shoreline retreat are important, so we aggregated the pixel data by shoreline reaches with a single orientation and oil fraction. A vector of the 2006 marsh shoreline was used as a baseline for generating transects every 100m using an onshore transect sampling algorithm. The sinuosity of the marsh shorelines and number of small marsh islands (< 1000m^2^) in the southern Barataria Basin resulted in frequently overlapping onshore transects and created shoreline reaches that were variable in length (S1 Fig). Where overlapping transects generated longshore reaches that were less than 15 m, the transects were manually removed, resulting in reaches that ranged in length from 15–172 m (S2 Table). We then examined land loss in relation to oil fractional cover along the segmented longshore reaches (N = 1443, 133 km of marsh shoreline) (S2 Table). The ArcGIS zonal statistics tool was used to calculate the area of land loss per reach over each time period, and to calculate the mean oil fractional cover over the same shoreline reaches. Finally, the ArcGIS spatial join tool was used to link all the reach attributes (shoreline reach length, land loss area for each time period and mean oil fraction) to a single shoreline vector file.

To account for the variable lengths of the created longshore reaches (15–172 m), we normalized the total land loss by the reach length to get a standardized loss rate (*slr*) in m yr^-1^:

slr=(al)t (1)

where *slr* is calculated as the land area loss (*a*) over the segmented longshore reach length (*l*) divided by the number of years between image acquisitions (*t*). The time intervals (*t*) between imaging dates were 3.4 (2006–2010), 3.6 (2010–2013) and 2.5 (2013–2016) years. We performed reach-level pair-wise T-tests, and then summarized the p-values for each oiling category to determine if post-oiling land loss rates were significantly different from the pre-oiling rates (Table 1).

Post-spill land loss rates were higher for all shoreline reaches, including non-oiled reaches, presumably due to normal erosion forces affecting all reaches, such as wave energy, currents, tides and sediment supply. Additionally, storm surge from Hurricane Isaac, which made landfall in the study area in August 2012, likely contributed to increased land losses during the first post-oiling period (2010–2013). Therefore, we estimated background loss rates (*blr*) for a given oiling category (*j*) for the post-oiling periods as:

$$blr_{j}=k\left(slr_{j}^{pre}\right)$$ (2)

where k is the ratio of *slr* for post-oil (*slr post*,*no*.*oil*) and pre-oil (*slr*, *pre*,*no*.*oil*) periods for non-oiled reaches (oil cover = 0):

$$k=\frac{slr_{post,\;no.oil}}{slr_{pre,no.oil}}$$ (3)

For instance, the post-oiling (2010–2013) change coefficient (k_*1*_ = 1.36) was calculated by dividing the post-oiling (2010–2013) period *slr* (0.36 m^2^ m^-1^ yr^-1^) by the pre-oiling *slr* (0.26 m^2^ m^-1^ yr^-1^) for non-oiled reaches. The oil-related loss rate (*olr*) is the difference between the observed *slr* and the *blr*. All three loss rates were converted into an area loss rate by multiplying the loss rate by the length of shoreline for each oiling category.

## Results

### General land loss patterns over the three periods

Sixty-two percent of the shoreline (N = 993; 82,446 m) exhibited no detectable oiling (i.e. oil cover = 0) from July 2010—April 2011 (Fig 1, Table 1, S2 Fig). Twenty-one percent of the shoreline had mean oil fractions greater than 50%, and the remaining 17% had mean oil fractions between 3 and 49% (Fig 1, Table 1). Shorter reaches (< 50m) could have a disproportionate effect on land loss rates along the shoreline, however, these reaches only accounted for 7% of the total shoreline length. Additionally, 91% of the shorter reaches (< 50m) were along non-oiled shorelines.

**Fig 2:**
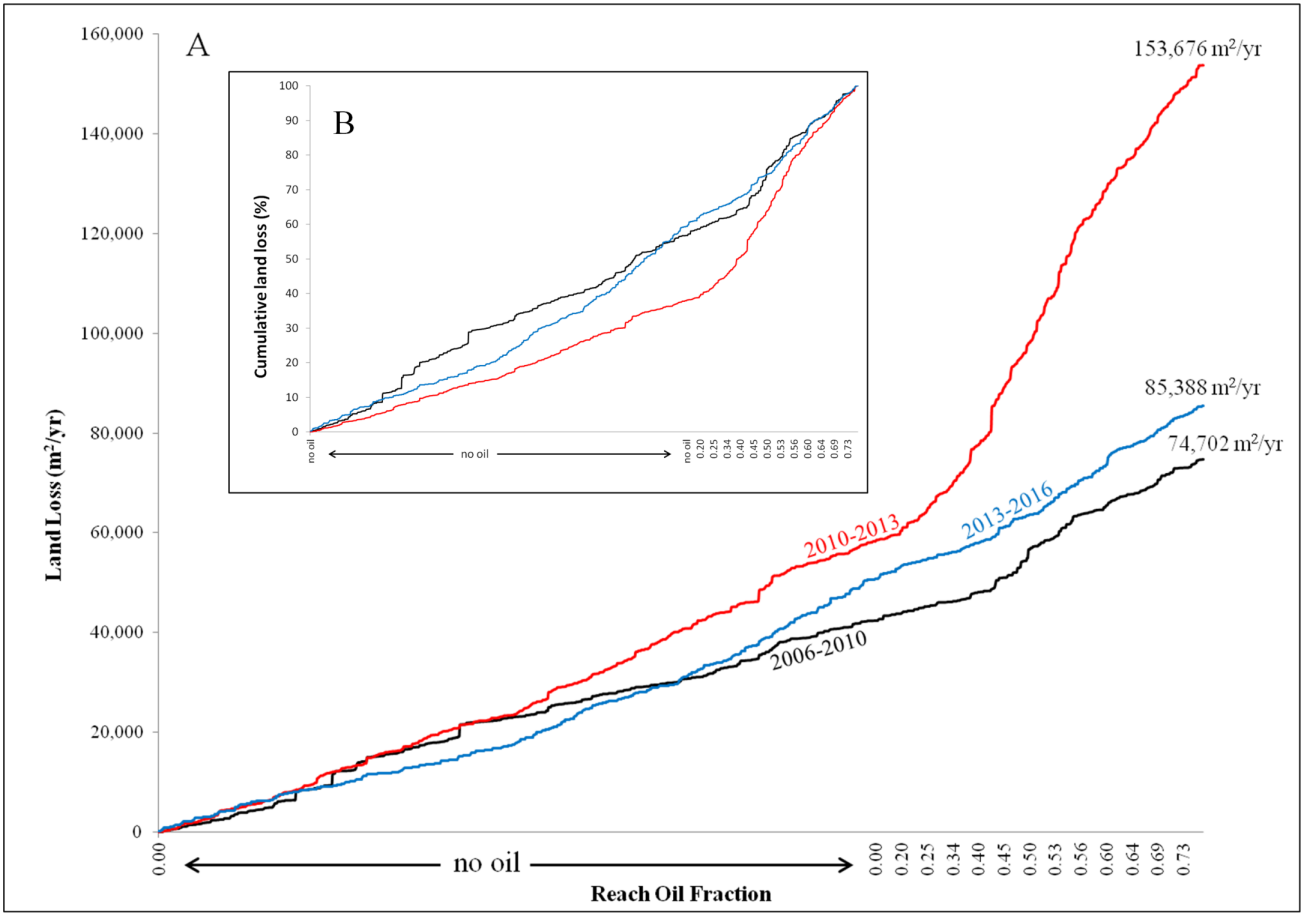
Cumulative land loss plots. Shows land losses in m^2^yr^-1^ (A) and percent of cumulative losses (B) over reaches with increasing oil fractions for the three time periods.

Total land loss during post-oiling period 1 (2010–2013) more than doubled the losses from the pre-oiling period (2006–2010) (Fig 2A), and then returned to near the pre-oiled rate in the post-oiling period 2 (2013–2016). Total land loss for all reaches increased from 74,702 m^2^ yr^-1^ (0.49 m^2^ m^-1^ yr^-1^) to 153,676 m^2^ yr^-1^ (1.44 m^2^ m^-1^ yr^-1^) from 2010–2013, and decreased to 85,388 m^2^ yr^-1^ (0.58 m^2^ m^-1^ yr^-1^) from 2013–2016 (Fig 2A). For non-oiled reaches (82,446 m), the *slr* was highest during the first post-oil period (2010–2013, 0.71 m^2^ m^-1^ yr^-1^), however, there were no statistical differences in *slr* between 2010–2013 and the pre-oiling period (0.53 m^2^ m^-1^ yr^-1^, p = 0.129) or the 2013–2016 period (0.63 m^2^ m^-1^ yr^-1^, p = 0.249) (Table 1). Even though the land loss rates for non-oiled reaches were highest during the 2010–2013 period, their relative contribution to the total losses were low (Fig 2B). Land loss within non-oiled reaches contributed to 38% (58,762 m^2^/yr) of the total land loss during 2010–2013, despite accounting for 62% of the shoreline length (Table 1). In comparison, land loss within non-oiled reaches accounted for 58% (43,348 m^2^ yr^-1^) and 61% (51,904 m^2^ yr^-1^) of the total land loss during the 2006–2010 and 2013–2016 periods, respectively (Table 1), which is comparable to the percent shoreline length, suggesting non-oiled and oiled shorelines had similar relative contributions to land loss in pre- and the second post-oiled periods (Fig 2B). Furthermore, there was no statistical difference observed for any reach oiling categories from pre-oiling and post-oiling period 2 (2013–2016), suggesting land loss had returned to background rates by the second post-oiling period (2013–2016) (Table 1).

### Land loss trajectories along oiled reaches

**Fig 3:**
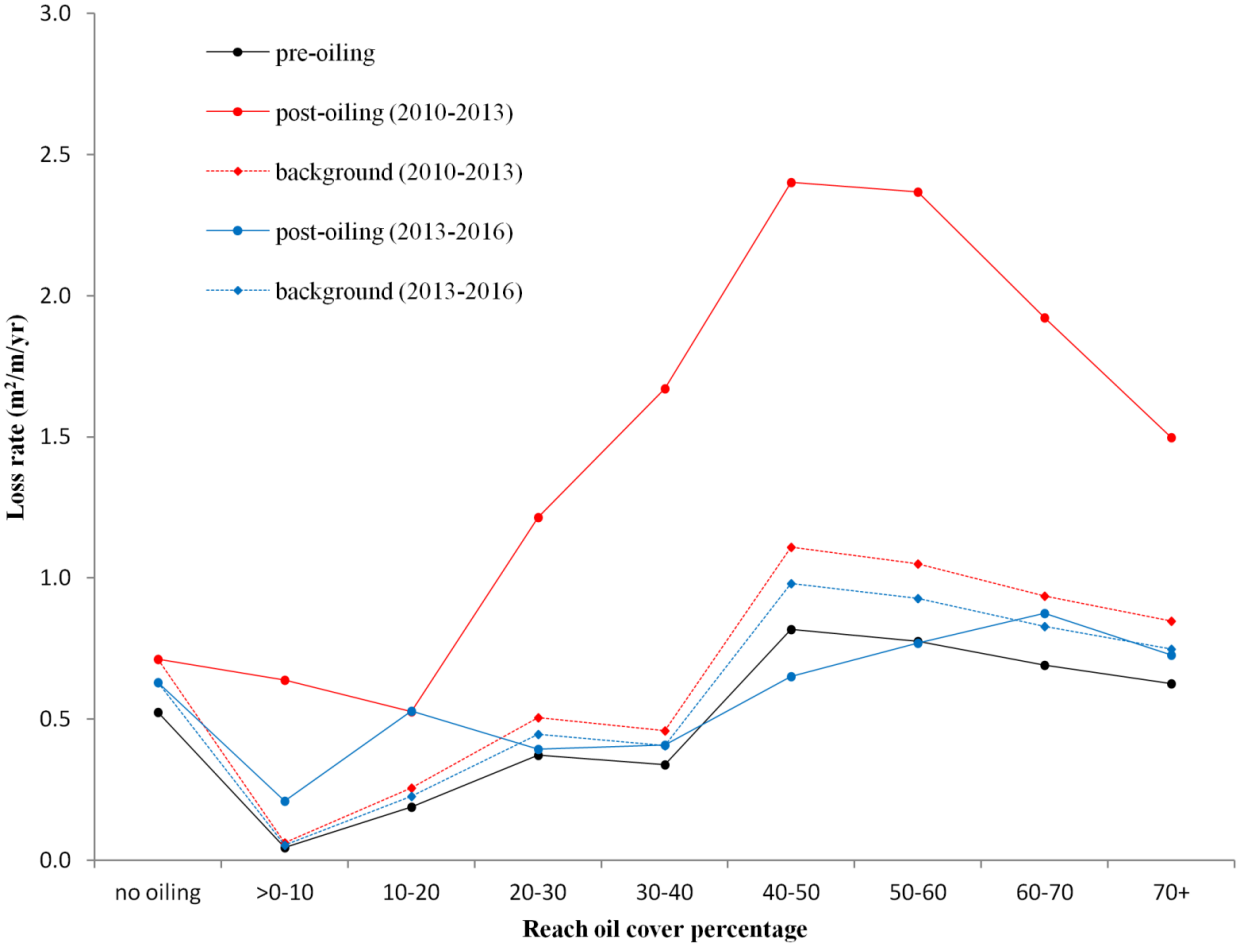
Land loss rates over reach oiling categories. Pre-oiling (2006–2010), post-oiling (2010–2013), post-oiling (2013–2016), and background land loss rates over reach oiling categories.

**Fig 4:**
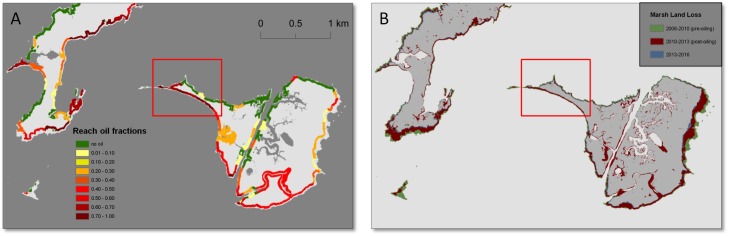
Maps of shoreline oiling category and corresponding land loss in Bay Jimmy (map location is shown in Fig 1). Map A shows shoreline zones and reach mean oil fractions, and map B shows marsh land loss along the same reaches over the three time periods. Narrow strip of the Bay Jimmy island (red box) is an area that experienced extensive oiling treatments for remediation.

Reaches with oiling, particularly mean oil fractions ≥20%, exhibited noticeably higher land loss rates during post-oiling period 1 (2010–2013) (Fig 3). Further, the trajectory of land loss rates during this period is significantly different than either the pre-oiling or post-oiling period 2 (2013–2016) (Figs 2 and 3, Table 1). The loss rates during the first post-oiling period are consistently and significantly higher (p < 0.05) than the pre-oiling period and post-oiling period 2 (2013–2016) for reaches with ≥20% oiling (Fig 3, Table 1), though land loss rates in post-oiling period 1 (2010–2013) do not increase monotonically with oiling and reached a maximum at 40–60% oiling. The decrease in loss rate for shorelines with >60% oiling may partly be a product of remediation efforts along the heaviest oiled shorelines as discussed in Zengel et al. [36]. For instance, the island in Bay Jimmy in Fig 4 (red box) received extensive treatment following oiling, including both mechanical and manual treatments, which may have contributed to suppressed shoreline erosion [36]. The average loss rates in post-oiling period 1 (2010–2013) for reaches with ≥20% oiling are more than three times the rates of both the pre-oiling and the post-oiling period 2 (Table 1). Reaches with ≥20% oiling (36% of shoreline length) accounted for 93,674 m^2^ yr^-1^ of land loss, or 62% of the total land loss for the post-oiling period 1 (2010–2013). In comparison, the land losses from the pre-oiling period (31,030 m^2^ yr^-1^, 42% of total loss) and post-oiling period 2 (2013–2016: 32,317 m^2^ yr^-1^, 38% of total loss) are more similar to the relative length of the shorelines. Reaches with ≥50% oiling contributed 37% of the land loss during the post-oiling period 1 (2010–2013), while accounting for 21% of the shoreline length (Table 1).

### Background rates and oil-related land losses

Mean background loss rates along the shoreline are 0.65 and 0.58 m^2^ m^-1^ yr^-1^ for oiled reaches, and range from 0.06–1.11 and 0.05–0.98 m^2^/m/yr over the two consecutive post-oiling periods (Fig 3). Background land loss area for oiled reaches are 42,635 and 37,650 m^2^/yr for the two post-oiled periods, accounting for 28% and 44% of the total losses (Table 1).

Total land losses along oiled reaches increased by 55%, or 52,521 m^2^ yr^-1^, in the first post-oil period (2010–2013), more than 80% of which are attributable to the 30–70% oiled reaches (Table 1). The background loss rate for post-oiling period 2 (2013–2016) accounts for all of the erosion observed. The estimated background loss rates were slightly higher than the observed loss rates for some reaches in the second post-oil period, which resulted in area loss estimates that were above the observed losses for the 40–60% oiled reaches (Fig 3, Table 1).

### Spatial patterns of land loss

**Fig 5:**
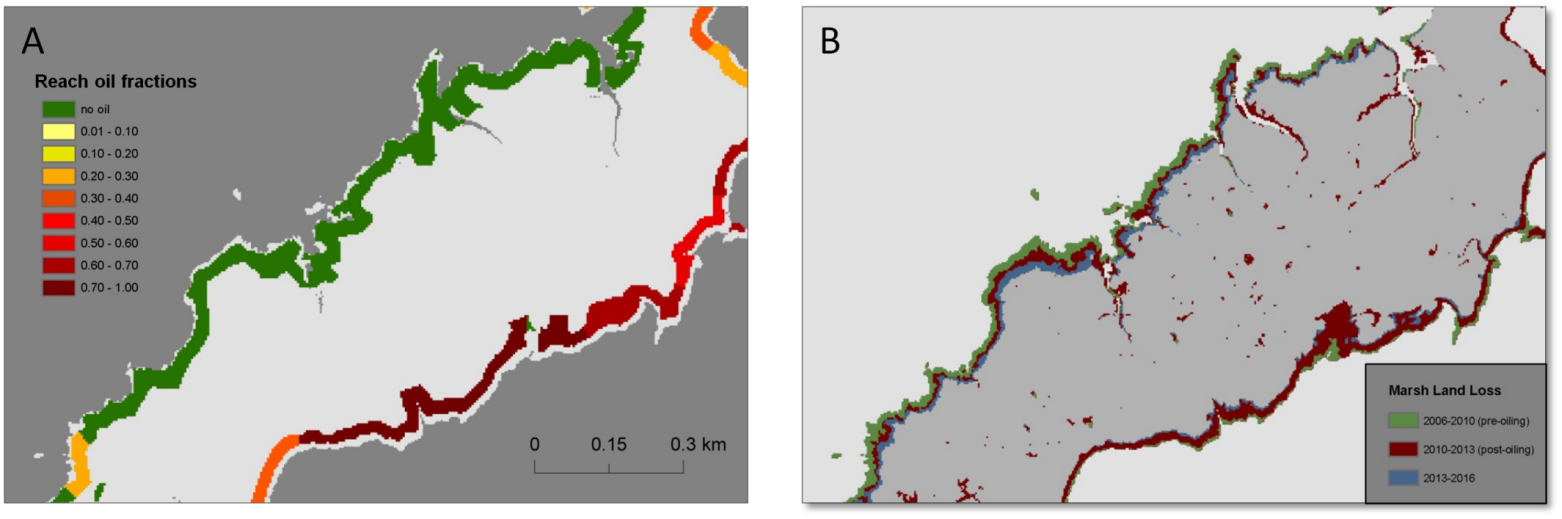
Maps of shoreline oiling category and corresponding land loss (map location is shown in Fig 1). Map A shows shoreline zones and reach mean oil fractions, and map B shows marsh land loss along the same reaches of northern Bay Jimmy over the three time periods.

**Fig 6:**
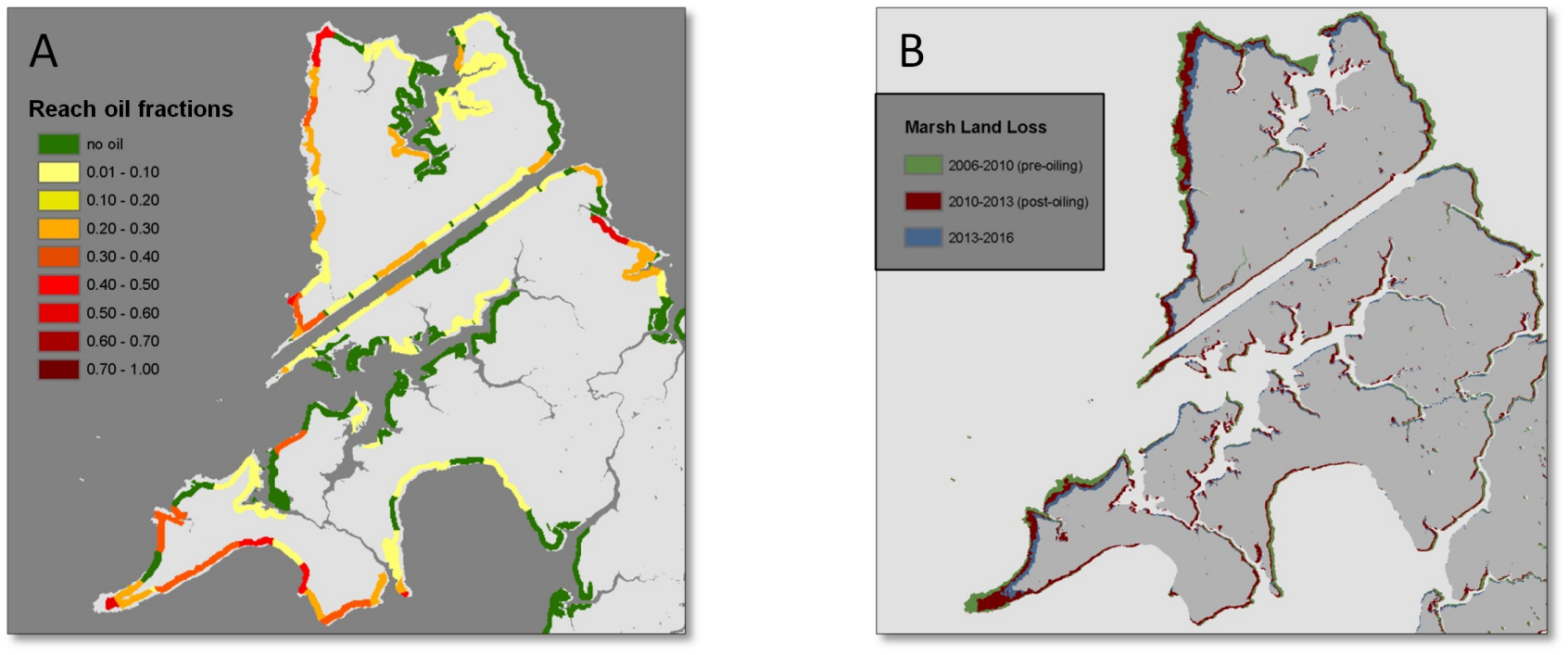
Maps of shoreline oiling category and corresponding land loss in Bay Batiste (map location is shown in Fig 1). Map A shows shoreline zones and reach mean oil fractions, and map B shows marsh land loss along the same reaches of eastern Bay Batiste over the three time periods.

**Fig 7:**
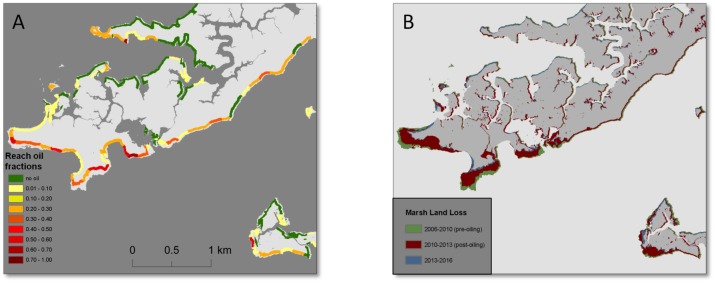
Maps of shoreline oiling category and corresponding land loss in Bay Batiste (map location is shown in Fig 1). Map A shows shoreline zones and reach mean oil fractions, and map B shows marsh land loss along the same reaches of southeastern Bay Batiste over the three time periods.

Two distinct spatial patterns are observed in the maps of progressive land loss over the three time periods (Figs 4–7). First, we observed substantial land loss along non-oiled, north facing shorelines. In Fig 5, non-oiled reaches (A) along the northern shoreline exhibit shoreline retreat and land loss over all three periods. A similar pattern of land loss is shown in Fig 6B, along the northeast facing non-oiled reach. Second, moderate to heavily oiled shorelines show the greatest losses, particularly in the post-oiling period 1 (2010–2013), and highest rates of land loss are predominantly along south and southeast facing shorelines as seen in Figs 4–7. In Figs 4, 5 and 7, the south to southeast facing shorelines exhibit heavy oiling (A), and extensive land loss from 2010–2013 (B). These reaches exhibited far less shoreline retreat and land loss during the pre-oiling and 2013–2016 time periods (Figs 4B, 5B and 7B).

## Discussion

### Land losses in historical context

The Mississippi River Delta, particularly the Terrebonne and Barataria basins, has among the highest land loss rates of any deltaic system in the United States [17], which is driven by both natural and anthropogenic forcings [21,23,55,56,57,58]. Vertical erosion processes have been attributed to canal dredging, river channelization, land subsidence and sea-level-rise [21,23,57,58], while wave energy has been the primary contributor to lateral erosion (i.e. marsh edge undercutting) [55,56,59]. Herein, we report overall land loss rates of 1.03% (2006–2010), 1.40% (2010–2013) and 1.23% (2013–2016) wetland area per year for non-oiled shoreline zones (i.e. ≤ 21m from marsh edge), which are consistent with earlier periods of land loss in Barataria Bay. Similar rates were reported for the 1956–1970 (0.70–1.2%) [15,18,60,61,62] and the 1990–2000 time periods (0.90%) [63]. Our findings are higher than the rates reported from 1933–1956 (0.20–0.37%) [17,18,61] and 2000–2010 (0.49%) [17], but less than the 1970–1990 peak loss period (1.90–2.04%) [18,61,63], which suggests that 2006–2016 was a period of intermediate rates of erosion from non-oiling related forces. The lower rates of land loss, during the 1933–1956 and 1990–2010 periods, are consistent with rates averaged over geological time scales that are attributable to sediment compaction and deep crustal loading [58]. The peak land loss rates during the 1970–1990 period are likely the direct result of accelerated subsidence from fluid extraction for oil and gas production, which increased in the Mississippi Delta during the 1960's and 1970's [58].

Our reported rates (i.e. 1.03%, 1.40% and 1.23% for the three periods) are somewhat higher than what Barras et al. [63] (0.90%) and Couvillion et al. [17] (0.49%) reported for the most recent pre-oil period (1990–2010). The observations of land loss conducted during these earlier studies used coarser imagery (Landsat: 30 m), which may account for some of the discrepancy in land loss rates. The difference in rates is more likely a result of our focus on near-shore marshes (i.e. ≤ 21m from marsh edge). Historically, interior marshes of Barataria Bay have comparatively low land loss rates [17], therefore, we expect that the percentage of total wetland loss would decrease to around the previously reported rates attributable to natural processes (i.e. 0.40–0.90% yr^-1^), if interior marshes were included in the analysis. A more appropriate comparison of non-oiled near-shore marsh loss rates is with the bay islands of Barataria Bay, due to their similar biogeomorphic profiles, which are comprised of low, relatively flat monotypic (i.e. *Spartina alterniflora* dominant) marsh platforms behind 30–50 cm natural levees at the marsh edge [42,43]. Our annual wetland loss rates post-oiling (2010–2013: 1.40% yr^-1^ and 2013–2016: 1.23% yr^-1^) are comparable to the marsh island area loss rates of 1.5–1.6% reported in Turner et al. [43] for non-oiled islands. Rates of shoreline retreat along non-oiled shorelines that are reported in our analysis (0.99–1.21 m yr^-1^) are also similar to the rates reported previously in specific study sites of Barataria Bay (0.80–1.38 m yr^-1^) [25,26,38].

### Impact of oiling on land loss trajectory

Several recent studies have used remote sensing techniques to assess the impacts of oiling on salt marsh vegetation [30,41,64,65,66] and marsh land loss [41,42,43,67]. Herein, we take a unique approach to quantifying the impact of oiling on reach-scale and bay-wide loss rates, while controlling for temporal variability in natural background erosion rates. The most notable results from our analysis are: 1) the differences in land loss trajectories reported for the first 3-years post-oiling (2010–2013) and the other two periods, and 2) the magnitude of land loss beyond background rates. The curves of cumulative land loss by oiled fraction are relatively similar for the pre-oiling period and 3–6 years after oiling (2013–2016), and exhibit no significant differences across all reaches (Fig 2, Table 1). There is a striking increase in land loss rates during the 2010–2013 period for all reaches with oiling ≥20% (Fig 3). As expected, the substantial increase in loss rates contributed to total land losses that are more than double the period before (2006–2010) or after (2013–2016) (Table 1).

Heavy oiling has complex and interactive effects on the structural and physiological traits of marsh macrophytes that likely influence recovery success [32,40,68]. Plant community composition (i.e. stem density, above- and belowground productivity) may influence residual oil concentrations and ecosystem response [7,31,33,40]. Most petroleum crude oils (e.g. south Louisiana crude) are nonionic, and therefore, associate more readily with organic particles [40]. Consequently, soil organic matter (SOM) in a marsh substrate impacts oil concentrations, and SOM content varies depending on plant species composition [7]. Lin and Mendelssohn [7] reported both higher SOM content and higher oil residual concentrations in plots dominated by *Spartina patens* than those dominated by *S*. *alterniflora*. In both field and mesocosm experiments, *S*. *alterniflora* has exhibited a greater recovery rate than *Juncus roemerianus*, indicating a higher tolerance limit for oil contamination [31,69]. Live aboveground biomass and stem density were about 10 times greater for *S*. *alterniflora* than *J*. *roemerianus* after 18 months under heavy oiling conditions [31]. Recently, Beland et al., [41] reported that only *S*. *alterniflora* dominated marshes were extensively degraded following the DWH spill, losing 15% (354,604 m2) cover along oiled shorelines, suggesting that marsh degradation may have been worse if the oil-impacted marshes were dominated by other species i.e. (*J*. *roemerianus* or *S*. *patens*).

For heavily oiled shorelines (>50% oil fraction), we report loss rates 2.7 times greater (2.1 times for all oiled reaches) than that of non-oiled shorelines for the first 3-years after oiling. This magnitude of impact from oiling is consistent with observations of land loss on Barataria Bay marsh islands from Turner et al. [43] and from site-specific studies [25,36], which have reported erosion rates at heavily oiled plots that were 2–3 times that of reference, non-oiled plots within 2 years of initial oiling. Our bay-wide results show that oiled shorelines experienced 2.1 times the loss rate of non-oiled shorelines over 3 years, which is in agreement with Turner et al. [43] observations of oiled island shorelines that were 2.0 times greater than non-oiled islands over 2.5 years. Accounting for bay-wide background land losses from natural processes (42,625 m^2^ yr^-1^), we determined 52,521 m^2^ yr^-1^ of land was lost due to oiling, increasing the land losses by 52% over the background rate.

Two marsh erosion processes, driven by heavy oiling, were presumably contributing to the accelerated rates of land loss that we observed. Exposure to heavy oiling obstructs critical, adaptive mechanisms for reducing oxygen stress in anoxic soils [40], and for controlling tissue salt (Na+ or Cl−) concentrations through osmotic adjustment [68]. Further, long-term (months to years) exposure to heavy residual oiling has resulted in reduced aboveground primary productivity and root matrix mortality, which are critical components of soil strength [70,71,72,73,74]. Consequently, above- and belowground plant loss and reductions in primary productivity have resulted in substrate instability and increased potential for shoreline erosion [25,26,36,69]. Early assessments following the DWH spill reported widespread vegetation mortality and deterioration of the aboveground vegetation structure and function at heavily oiled sites [25,31,36], resulting in slow rates of recovery with aboveground biomass reaching only 50% of that in reference sites after 3.5 years [36,69], and accelerated surface subsidence (vertical erosion) [25,69]. Further, heavily oiling in marsh soils have also resulted in losses of belowground biomass, weakening soil shear strength and accelerating the undercutting along marsh edges [26,36].

The influence of other factors, including: oiling characteristics [7,22,33,40,75,76,77,78,79,80,81] and treatment methods [36,82,83,84,85], environmental stressors (e.g. salinity, flooding, nutrients, predation) [2,86,87,88], as well as complex and interactive marsh biogeochemical processes [89,90,91,92] make attributing the landscape-scale progression of marsh deterioration and land loss to oiling difficult [43,93,94]. We attempt to control for the influence of these factors on land loss by calculating reach-scale background rates over the three periods between image acquisition dates.

This analysis is the first to show that land loss rates returned to pre-oiling levels within 3–6 years after oiling, and that no significant differences in land loss rates are exhibited for any oiling category between the pre-oiling and latter post-oiling periods (*p* ≥ 0.095) (Table 1). Land loss was higher in the second post-oiling period (2013–2016) compared with pre-oiling, but non-oiled reaches accounted for 81% of this increase, which suggests that any increases in land loss related to oiling is negligible from three to six years after initial contamination.

We provided a landscape-scale, bay-wide quantification of land loss, while documenting the return to background erosion rates 3–6 years after oiling. Yet, several obvious questions remain unaddressed, such as what is the relative importance of lateral erosional forces from wave action in comparison to vertical forces (i.e. reduced sediment accretion and subsidence) in Barataria Bay? Wilson and Allison, [38] estimated that 25% of wetland losses in southeastern Louisiana are due to lateral erosion from wave action, particularly along shorelines exposed to long fetches and predominant direction of wave approach. Over the last century, shoreline erosion has likely accelerated in Barataria Bay as the conversion from marsh platform to open water has increased the fetch and wave energy on exposed marsh edges [38]. Oiling in Barataria Bay occurred most frequently on the southside of landmasses and marsh islands [29,30], and we anticipated oil distribution might be correlated with pre-oiling shoreline erosion rates, due to the strong influence of currents, wave energy and tides on both processes [43]. Yet, the land loss along non-oiled shorelines was substantial and relatively similar during all three time periods, and these loss rates were at least equal to the rates of oiled reaches during the pre-oiling period. Future research will need to explicitly investigate the compounding role wave action has on lateral erosion and overall land loss rates.

The impact the use of marsh treatments had on bay-wide land losses is still largely unknown. Clean-up and treatment efforts affect the recovery process, both positively and negatively [82,83]. Aggressive treatment strategies, including the use of large cleanup crews or heavy machinery, have delayed marsh recovery or increased degradation by trampling live vegetation and churning oil into underlying sediments [83,84,85]. Conversely, less intrusive treatments, which include the use of sorbents, bioremediation, and restricted cutting, have been shown to accelerate the rates of recovery [79,82]. Two years after the DWH spill, Zengel et al. [36] reported both mechanical and manual treatments exhibited greater improvements in oiling conditions and vegetation characteristics than the natural recovery (reference sites). However, mechanical treatments increased oil mixing in soils and accelerated shoreline erosion [36]. Other analysis has indicated that shoreline erosion was similar on both treated and non-treated shorelines [42]. Due to the potential impact of treatments, the location and treatment type should be regarded as a factor in a future analysis of marsh responses to oiling.

Finally, we showed that land losses increased significantly for the first three years after oiling, followed by a return to background erosion levels after three years. To date, this process of returning to background rates of erosion remains unexplained, and should be addressed in future research. Hester et al. [94] reported evidence of vegetation stress (chlorosis), lower stem densities and productivity for the first 2.5 years, but few significant impacts to plant aboveground productivity (for plots that did not erode away) for heavily oiled plots 3.5 years after the DWH spill, which may suggest that vegetation recovery and presumably substrate stability had returned to heavily oiled marshes that were not eroded in the first three years. Conversely, Lin et al. [69] reported that belowground biomass (0–12 cm) at heavily oiled plots was 76% less than reference sites after 3.5 years, which may suggest substrate instability is an ongoing problem. Further research is required on the interactions among belowground biomass recovery, resistance to wave-driven erosion, the sequence and magnitude of wave events, and subsequent shoreline erosion.

## Conclusion

We examined the relative land loss rates of oiled shoreline reaches compared to non-oiled reaches of Barataria Bay over three consecutive time periods. Oiling increased total land losses by 52,521 m^2^ yr^-1^, in the first post-oil period (2010–2013), more than 80% of which are attributable to the 30–70% oiled reaches. No statistical difference was observed for any reach oiling categories from pre-oiling and post-oiling period 2 (2013–2016). Oiling increased land loss by more than 50%, but land loss rates returned to background levels within 3–6 years after oiling, suggesting that oiling results in a large but temporary increase in shoreline loss.

We attempted to control for effect of other erosional forces (i.e. wave energy, variability in landscape position and geomorphic profile) on land loss by calculating the background rates (*blr*) derived from pre-oiling land loss patterns and post-oiling land loss in non-oiled reaches. Our calculation of *blr* assumes that the ratio between the loss rate in the pre-oil and post-oil period is the same under non-oiled conditions for all shoreline orientations and locations in the bay. Wave modeling could be included in future analysis to control for changes in shoreline orientations, wind direction and fetch between periods.

This study does not examine the relative contributions of oiling as it relates to other drivers of land loss, or efforts to suppress shoreline erosion following oil contamination. For instance, Rangoonwala et al. [42] showed that storm surge from Hurricane Isaac (August 2012) contributed to a 2.5x increase in the shoreline length that experienced lateral recession of >12 m over a 4-month period. Further, Zengel et al. [36] documented significant differences in the ecological responses of oiled marshes that received manual and mechanical treatments, and those not receiving remediation. Going forward, a spatially explicit model could determine the relative importance of multiple factors, including oiling (oil fractional cover), wave energy (significant wave height, period and length), vegetation composition (green and non-photosynthetic vegetation, aboveground biomass) and treatment type (manual, mechanical and no remediation) on predicting land loss. The results from this analysis, along with a spatially-explicit predictive model, would help inform future management decisions regarding coastal wetland ecosystems.

## Supporting information

S1 Table Descriptions of the images used in the land loss analysis. (TIF)

S2 Table Individual reach length, land loss and oil fraction data for the three time periods. (XLSX)

S1 Fig Shoreline reaches and onshore zones. Examples of shoreline zones (green) and the onshore transects (black lines) that were used to segment the shoreline into reaches. For shorelines that were relatively linear (A), the transects segmented the shoreline into 100m reaches. However, for undulating shorelines (B), onshore transect direction was irregular and at times overlapping, creating shorter onshore reaches. Additionally, transect generation on small islands (C) commonly resulted in inconsistent reach lengths, and in some cases, required manual removal of selective transects.
(TIF)

S2 Fig Map time-series showing wetland change from 2006–2016. (ZIP)

## References

[1] Turner R.E. (1976). Geographic variations in salt marsh macrophyte production: a review. Contrib Mar Sci 20:47–68.

[2] Pennings S.C., Bertness M.D. (2001). Salt marsh communities In: Bertness M.D. G SD, Hay ME, editors. Marine Community Ecology: Sinauer Associates.

[3] Gedan K.B., Silliman B.R., and Bertness M.D. (2009). Centuries of human-driven change in salt marsh ecosystems. *Annual Review of Marine Science*, 1, 117–141. doi: 10.1146/annurev.marine.010908.163930 21141032 10.1146/annurev.marine.010908.163930

[4] Niering W.A., Warren R.S., Weymouth C.G. (1977). Our dynamic tidal marshes: vegetation changes as revealed by peat analysis. Connecticut Arboret Bull 22:2–12.

[5] Smart R.M., & Barko J.W. (1978). Influence of sediment salinity and nutrients on the physiological ecology of selected salt marsh plants. *Estuarine and Coastal Marine Science*, 7(5), 487–495.

[6] Pezeshki S.R., & Laune R.D. (1993). Effect of crude oil on gas exchange functions of Juncus roemerianus and Spartina alterniflora. *Water*, *Air*, *& Soil Pollution*, 68(3), 461–468.

[7] Lin Q., & Mendelssohn I.A. (1996). A comparative investigation of the effects of south Louisiana crude oil on the vegetation of fresh, brackish and salt marshes. *Marine Pollution Bulletin*, 32(2), 202–209.

[8] Weis J.S., & Weis P. (2004) Metal uptake, transport and release by wetland plants: implications for phytoremediation and restoration. Environ Int 30:685–700b. doi: 10.1016/j.envint.2003.11.002 15051245 10.1016/j.envint.2003.11.002

[9] Deegan L., Kennedy H., and Neill C. (1984). Natural factors and human modifications contributing to marsh loss in Louisiana’s Mississippi River deltaic plain Environ. Manag. 8 519–27.

[10] van de Koppel J., van der Wal D., Bakker J.P., & Herman P.M. (2005). Self-organization and vegetation collapse in salt marsh ecosystems. *The American Naturalist*, 165(1), E1–E12. doi: 10.1086/426602 15729634 10.1086/426602

[11] Stedman S.M., & Dahl T.E. (2008). *Status and trends of wetlands in the coastal watersheds of the eastern United States*, *1998 to 2004*. National Oceanic and Atmospheric Administration, National Marine Fisheries Service.

[12] Barrett B. (1970). Water measurements of coastal Louisiana: Louisiana Wildlife and Fisheries Commission, Division of Oysters, Water Bottoms and Seafood, p. 196.

[13] Gagliano S.M., Kwon H.J., & Van Beek J.L. (1970). Deterioration and restoration of coastal wetlands. *Coastal Engineering Proceedings*, 1(12).

[14] Chabreck R.H. (1972). Vegetation, water and soil characteristics of the Louisiana coastal region.

[15] Adams R.D., Barrett B.B., Blackmon J.H., Gane B.W., & McIntire W.G. (1976). Barataria Basin: Geologic processes and framework. Sea Grant Publ. La. State Univ. Cent. Wetl. Resour, (8), 111.

[16] Craig N.J., Turner R.E., & Day J.W. Jr (1979). Land loss in coastal Louisiana (USA). *Environmental Management*, 3(2), 133–144.

[17] Couvillion, B.R., Barras, J.A., Steyer, G.D., Sleavin, W., Fischer, M., Beck, H., et al. (2011). Land area change in coastal Louisiana from 1932 to 2010: U.S. Geological Survey Scientific Investigations Map 3164, scale 1:265,000, 12 p. pamphlet.

[18] Evers D.E., Gosselink J.G., Sasser C.E., & Hill J.M. (1992). Wetland loss dynamics in southwestern Barataria basin, Louisiana (USA), 1945–1985. *Wetlands Ecology and Management*, 2(3), 103–118.

[19] FitzGerald, D., Kulp, M., Hughes, Z., Georgiou, I., Miner, M., Penland, S., & Howes, N. (2007). Impacts of rising sea level to backbarrier wetlands, tidal inlets, and barrier islands: Barataria Coast, Louisiana. In Proceedings of Coastal Sediments (Vol. 7, pp. 179–1192).

[20] Britsch L.D., & Dunbar J.B. (1993). Land loss rates: Louisiana coastal plain. *Journal of coastal research*, 324–338.

[21] Turner R.E. (2011). Beneath the salt marsh canopy: loss of soil strength with increasing nutrient loads. *Estuaries and Coasts*, 34(5), 1084–1093.

[22] Hester M.W., & Mendelssohn I.A. (2000). Long-term recovery of a Louisiana brackish marsh plant community from oil-spill impact: Vegetation response and mitigating effects of marsh surface elevation. *Marine Environmental Research*, 49(3), 233–254. 11285728 10.1016/s0141-1136(99)00071-9

[23] Ko J.-Y., & Day J.W. (2004). A review of ecological impacts of oil and gas development on coastal ecosystems in the Mississippi Delta. *Ocean & Coastal Management*, 47(11–12), 597–623.

[24] Culbertson J.B., Valiela I., Pickart M., Peacock E.E., & Reddy C.M. (2008). Long-term consequences of residual petroleum on salt marsh grass. *Journal of Applied Ecology*, 45(4), 1284–1292.

[25] Silliman B.R., van de Koppel J., McCoy M.W., Diller J., Kasozi G.N., Earl K., et al (2012). Degradation and resilience in Louisiana salt marshes after the BP—Deepwater Horizon oil spill. *Proceedings of the National Academy of Sciences*, 109 (28), 11234–11239. 10.1073/pnas.1204922109 PMC3396483 22733752

[26] McClenachan G., Eugene Turner R., & Tweel A.W. (2013). Effects of oil on the rate and trajectory of Louisiana marsh shoreline erosion. *Environmental Research Letters*, 8(4), 044030.

[27] Lehr, B., Nristol, S., & Possolo, A. (2010). Oil Budget Calculator—Deepwater Horizon, technical documentation: a report to the National Incident Command. Coastal Response Res. Cent.

[28] Michel J., Owens E.H., Zengel S., Graham A., Nixon Z., Allard T., et al (2013). Extent and degree of shoreline oiling: Deepwater Horizon oil spill, Gulf of Mexico, USA. *PloS one*, 8(6), e65087 doi: 10.1371/journal.pone.0065087 23776444 10.1371/journal.pone.0065087 PMC3680451

[29] Kokaly R.F., Couvillion B.R., Holloway J.M., Roberts D.A., Ustin S.L., Peterson S.H., et al (2013). Spectroscopic remote sensing of the distribution and persistence of oil from the Deepwater Horizon spill in Barataria Bay marshes. *Remote Sensing of Environment*, 129, 210–230.

[30] Khanna S., Santos M.J., Ustin S.L., Koltunov A., Kokaly R.F., & Roberts D.A. (2013). Detection of salt marsh vegetation stress and recovery after the deepwater horizon oil spill in barataria bay, Gulf of Mexico using AVIRIS data. *PloS one*, 8(11), e78989 doi: 10.1371/journal.pone.0078989 24223872 10.1371/journal.pone.0078989 PMC3818498

[31] Lin Q., & Mendelssohn I.A. (2012). Impacts and recovery of the Deepwater Horizon oil spill on vegetation structure and function of coastal salt marshes in the northern Gulf of Mexico. *Environmental science & technology*, 46(7), 3737–3743. 22369124 10.1021/es203552p

[32] Pezeshki S.R., & De Laune R.D. (1993). Effect of crude oil on gas exchange functions of Juncus roemerianus and Spartina alterniflora. *Water*, *Air*, *and Soil Pollution*, 68(3–4), 461–468.

[33] DeLaune R.D., Pezeshki S.R., Jugsujinda A., & Lindau C.W. (2003). Sensitivity of US Gulf of Mexico coastal marsh vegetation to crude oil: Comparison of greenhouse and field responses. *Aquatic Ecology*, 37(4), 351–360.

[34] Gabet E.J. (1998). Lateral migration and bank erosion in a saltmarsh tidal channel in San Francisco Bay, California. *Estuaries*, 21(4), 745–753.

[35] Michel E.R., & Kirchner J.W. (2002). Effects of wet meadow riparian vegetation on streambank erosion. 2. Measurements of vegetated bank strength and consequences for failure mechanics. *Earth Surface Processes and Landforms*, 27(7), 687–697.

[36] Zengel S., Bernik B.M., Rutherford N., Nixon Z., & Michel J. (2015). Heavily oiled salt marsh following the Deepwater Horizon oil spill, ecological comparisons of shoreline cleanup treatments and recovery. *PloS one*, 10(7), e0132324 doi: 10.1371/journal.pone.0132324 26200349 10.1371/journal.pone.0132324 PMC4511762

[37] Hershner C., & Lake J. (1980). Effects of chronic oil pollution on a salt-marsh grass community. *Marine Biology*, 56(2), 163–173.

[38] Wilson C.A., & Allison M.A. (2008). An equilibrium profile model for retreating marsh shorelines in southeast Louisiana. *Estuarine*, *Coastal and Shelf Science*, 80(4), 483–494.

[39] Peterson S.H., Roberts D.A., Beland M., Kokaly R.F., & Ustin S.L. (2015). Oil detection in the coastal marshes of Louisiana using MESMA applied to band subsets of AVIRIS data. *Remote Sensing of Environment*, 159, 222–231.

[40] Pezeshki S.R., Hester M.W., Lin Q., & Nyman J.A. (2000). The effects of oil spill and clean-up on dominant US Gulf coast marsh macrophytes: a review. *Environmental pollution*, 108(2), 129–139. 15092943 10.1016/s0269-7491(99)00244-4

[41] Beland M., Roberts D.A., Peterson S.H., Biggs T.W., Kokaly R.F., Piazza S., et al (2016). Mapping changing distributions of dominant species in oil-contaminated salt marshes of Louisiana using imaging spectroscopy. *Remote Sensing of Environment*, 182, 192–207.

[42] Rangoonwala A., Jones C.E., & Ramsey E. (2016). Wetland shoreline recession in the Mississippi River Delta from petroleum oiling and cyclonic storms. *Geophysical Research Letters*.

[43] Turner R.E., McClenachan G., & Tweel A.W. (2016). Islands in the oil: Quantifying salt marsh shoreline erosion after the Deepwater Horizon oiling. *Marine Pollution Bulletin*, 110(1), 316–323. doi: 10.1016/j.marpolbul.2016.06.046 27349381 10.1016/j.marpolbul.2016.06.046

[44] Penland S., & Ramsey K.E. (1990). Relative sea-level rise in Louisiana and the Gulf of Mexico: 1908–1988. *Journal of Coastal Research*, 323–342.

[45] Day J.W., Britsch L.D., Hawes S.R., Shaffer G.P., Reed D.J., & Cahoon D. (2000). Pattern and process of land loss in the Mississippi Delta: a spatial and temporal analysis of wetland habitat change. *Estuaries*, 23(4), 425–438.

[46] Li C., White J.R., Chen C., Lin H., Weeks E., Galvan K., & Bargu S. (2011). Summertime tidal flushing of Barataria Bay: Transports of water and suspended sediments. *Journal of Geophysical Research*: *Oceans*, 116(C4).

[47] Hatton R.S., DeLaune R.D., & Patrick W.E.I. Jr (1983). Sedimentation, accretion, and subsidence in marshes of Bar&aria Basin, Louisiana1. *Limnol*. *Oceanogr*, 28(3), 494–502.

[48] Chuang W.S., & Wiseman W.J. (1983). Coastal sea level response to frontal passages on the Louisiana-Texas shelf. *Journal of Geophysical Research*: *Oceans*, 88(C4), 2615–2620.

[49] Visser J.M., Sasser C.E., Chabreck R.H., & Linscombe R.G. (1998). Marsh vegetation types of the Mississippi River deltaic plain. *Estuaries*, 21(4), 818–828.

[50] Roth K.L., Dennison P.E., & Roberts D.A. (2012). Comparing endmember selection techniques for accurate mapping of plant species and land cover using imaging spectrometer data. *Remote Sensing of Environment*, 127, 139–152.

[51] Somers B., Delalieux S., Verstraeten W.W., Van Aardt J.A.N., Albrigo G.L., & Coppin P. (2010). An automated waveband selection technique for optimized hyperspectral mixture analysis. *International Journal of Remote Sensing*, 31(20), 5549–5568.

[52] Somers B., Delalieux S., Stuckens J., Verstraeten W.W., & Coppin P. (2009). A weighted linear spectral mixture analysis approach to address endmember variability in agricultural production systems. *International Journal of Remote Sensing*, 30(1), 139–147.

[53] Jensen J.R., Cowen D.J., Althausen J.D., Narumalani S., & Weatherbee O. (1993). The detection and prediction of sea level changes on coastal wetlands using satellite imagery and a geographic information system. *Geocarto International*, 8(4), 87–98.

[54] Dobson J.E., Bright E.A., Ferguson R.L., Field D.W., Wood L.L., Haddad K.D., et al (1995). *NOAA Coastal Change Analysis Program (C-CAP)*: *guidance for regional implementation* (p. 92). US Department of Commerce, National Oceanic and Atmospheric Administration, National Marine Fisheries Service.

[55] DeLaune R.D., Nyman J.A., & Patrick W.H. Jr (1994). Peat collapse, ponding and wetland loss in a rapidly submerging coastal marsh. *Journal of Coastal Research*, 1021–1030.

[56] Nyman J.A., Carloss M., DeLaune R.D., & Patrick W.H. Jr (1994). Erosion rather than plant dieback as the mechanism of marsh loss in an estuarine marsh. *Earth Surface Processes and Landforms*, 19(1), 69–84.

[57] Day J.W., Boesch D.F., Clairain E.J., Kemp G.P., Laska S.B., Mitsch W.J., et al (2007). Restoration of the Mississippi Delta: lessons from hurricanes Katrina and Rita. *science*, 315(5819), 1679–1684. doi: 10.1126/science.1137030 17379799 10.1126/science.1137030

[58] Morton R.A., & Bernier J.C. (2010). Recent subsidence-rate reductions in the Mississippi Delta and their geological implications. *Journal of Coastal Research*, 555–561.

[59] Sasser C.E., Gosselink J.G., Swenson E.M., & Evers D.E. (1995). Hydrologic, vegetation, and substrate characteristics of floating marshes in sediment-rich wetlands of the Mississippi river delta plain, Louisiana, USA. *Wetlands Ecology and Management*, 3(3), 171–187.

[60] Gagliano S.M., Ashby F.A., & Guidry J. (1981). *Special report on marsh deterioration and land loss in the deltaic plain of coastal Louisiana*. Coastal Environments, Incorporated.

[61] Sasser C.E., Dozier M.D., Gosselink J.G., & Hill J.M. (1986). Spatial and temporal changes in Louisiana's Barataria Basin marshes, 1945–1980. *Environmental Management*, 10(5), 671–680.

[62] Turner R.E. (1990). Landscape development and coastal wetland losses in the northern Gulf of Mexico. *American Zoologist*, 30(1), 89–105.

[63] Barras J., Beville S., Britsch D., Hartley S., Hawes S., Johnston J., et al (2003). *Historical and projected coastal Louisiana land changes*: *1978–2050* (p. 39p). United States Geological Survey.

[64] Mishra D.R., Cho H.J., Ghosh S., Fox A., Downs C., Merani P.B., et al (2012). Post-spill state of the marsh: Remote estimation of the ecological impact of the Gulf of Mexico oil spill on Louisiana Salt Marshes. *Remote Sensing of Environment*, 118, 176–185.

[65] Ramsey E., Rangoonwala A., & Jones C.E. (2016). Marsh canopy structure changes and the Deepwater Horizon oil spill. *Remote Sensing of Environment*, 186, 350–357.

[66] Shapiro K., Khanna S., & Ustin S.L. (2016). Vegetation Impact and Recovery from Oil-Induced Stress on Three Ecologically Distinct Wetland Sites in the Gulf of Mexico. *Journal of Marine Science and Engineering*, 4(2), 33.

[67] Khanna S., Santos M.J., Koltunov A., Shapiro K.D., Lay M., & Ustin S.L. (2017). Marsh Loss Due to Cumulative Impacts of Hurricane Isaac and the Deepwater Horizon Oil Spill in Louisiana. *Remote Sensing*, 9(2), 169.

[68] Hester M.W., Mendelssohn I.A., & McKee K.L. (2001). Species and population variation to salinity stress in Panicum hemitomon, Spartina patens, and Spartina alterniflora: morphological and physiological constraints. *Environmental and Experimental Botany*, 46(3), 277–297.

[69] Lin Q., Mendelssohn I.A., Graham S.A., Hou A., Fleeger J.W., & Deis D.R. (2016). Response of salt marshes to oiling from the Deepwater Horizon spill: Implications for plant growth, soil surface-erosion, and shoreline stability. *Science of the Total Environment*, 557, 369–377. doi: 10.1016/j.scitotenv.2016.03.049 27016685 10.1016/j.scitotenv.2016.03.049

[70] Van Eerdt M.M. (1985). Salt marsh cliff stability in the Oosterschelde. *Earth Surface Processes and Landforms*, 10(2), 95–106.

[71] Michel E.R., & Kirchner J.W. (2002). Effects of wet meadow riparian vegetation on streambank erosion. 2. Measurements of vegetated bank strength and consequences for failure mechanics. *Earth Surface Processes and Landforms*, 27(7), 687–697.

[72] Feagin R.A., Lozada-Bernard S.M., Ravens T.M., Möller I., Yeager K.M., & Baird A.H. (2009). Does vegetation prevent wave erosion of salt marsh edges?. *Proceedings of the National Academy of Sciences*, 106(25), 10109–10113. 10.1073/pnas.0901297106 PMC2694034 19509340

[73] Francalanci S., Bendoni M., Rinaldi M., & Solari L. (2013). Ecomorphodynamic evolution of salt marshes: Experimental observations of bank retreat processes. *Geomorphology*, 195, 53–65.

[74] Wu T.H. (2013). Root reinforcement of soil: review of analytical models, test results, and applications to design. *Canadian Geotechnical Journal*, 50(3), 259–274.

[75] Lin Q., Mendelssohn I.A., Suidan M.T., Lee K., & Venosa A.D. (2002). The dose-response relationship between No. 2 fuel oil and the growth of the salt marsh grass, Spartina alterniflora. *Marine Pollution Bulletin*, 44(9), 897–902. 12405214 10.1016/s0025-326x(02)00118-2

[76] Alexander, S.K., & Webb, J.W. (1987). Relationship of Spartina alterniflora growth to sediment oil content following an oil spill. Proceeding of the 1987 Oil Spill Conference. American Petroleum Institute, Washington, DC, pp. 445–449.

[77] Mendelssohn I.A., Hester M.W., Sasser C., & Fischel M. (1990). The effect of Louisiana crude oil discharge from a pipeline break on the vegetation of a southeast Louisiana brackish marsh. Oil Chem. Pollut. 7, 1–15.

[78] Hoff, R.Z., Shigenaka, G., & Henry, C.B. (1993). Saltmarsh recovery from a crude oil spill: vegetation, oil weathering, and response. Proceeding of the 1993 International Oil Spill Conference, pp. 307–311.

[79] Lin Q., & Mendelssohn I.A. (2009). Potential of restoration and phytoremediation with Juncus roemerianus for diesel-contaminated coastal wetlands. Ecol. Eng. 35, 85–91.

[80] Culbertson J.B., Valiela I., Pickart M., Peacock E.E., & Reddy C.M., 2008 Long-term consequences of residual petroleum on salt marsh grass. J.Appl. Ecol. 45, 1284–1292.

[81] Mendelssohn I.A., Anderson G.L., Baltz D., Caffey R., Carman K.R., Fleeger J.W., et al (2012). Oil impacts to coastal wetland: implications for the Mississippi River delta ecosystem after the Deepwater Horizon oil spill. Biosciences 62, 562–574.

[82] Sell, D., Conway, L., Clark, T., Picken, G. B., Baker, J. M., Dunnet, G. M., et al. (1995). Scientific criteria to optimize oil spill cleanup. In International Oil Spill Conference (Vol. 1995, No. 1, pp. 595–610). American Petroleum Institute.

[83] Hoff, R. (1995). Responding to oil spills in coastal marshes: The fine line between help and hindrance. HAZMAT Report 96–1. Hazardous Materials Response and Assessment Division, NOAA, Seattle, WA, 17 pp.

[84] Baker, J.M., Guzman, L.M., Bartlett, P.D., Little, D.I., Wilson, C.M. (1993). Long-term fate and effects of untreated thick oil deposits on salt marshes. Proceedings, 1993 International Oil Spill Conference, American Petroleum Institute, Washington, D.C., pp. 395–399.

[85] Zengel, S. & Michel, J. (2013). Deepwater Horizon Oil Spill: Salt Marsh Oiling Conditions, Treatment Testing, and Treatment History in Northern Barataria Bay, Louisiana (Interim Report October 2011). U.S. Dept. of Commerce, NOAA Technical Memorandum NOS OR&R 42. Seattle, WA: Emergency Response Division, NOAA. 74 pp.

[86] Bertness M.D., & Shumway S.W. (1993). Competition and facilitation in marsh plants. *The American Naturalist*, 142(4), 718–724. doi: 10.1086/285567 19425967 10.1086/285567

[87] Silliman B.R., & Bertness M.D. (2002). A trophic cascade regulates salt marsh primary production. Proc. Natl. Acad. Sci. USA 99, 10500–10505. doi: 10.1073/pnas.162366599 12149475 10.1073/pnas.162366599 PMC124954

[88] Pennings S.C., Grant M.B., & Bertness M.D. (2005). Plant zonation in low-latitude salt marshes: disentangling the roles of flooding, salinity and competition. *Journal of ecology*, 93(1), 159–167.

[89] Atlas R.M., Stoeckel D.M. Faith S.A. & Minard-Smith A. (2015). Oil biodegradation and oil-degrading microbial populations in marsh sediments impacted by oil from the Deepwater Horizon well blowout. *Environmental Science & Technology* 49:8,356–8,366. 10.1021/acs.est.5b00413 26091189

[90] Marton J.M., Roberts B.J., Bernhard A.E., & Giblin A.E. (2015). Spatial and temporal variability of nitrification potential and ammonia-oxidizer abundances in louisiana salt marshes. *Estuaries and coasts*, 38(6), 1824–1837.

[91] Bernhard A.E., Sheffer R., Giblin A.E., Marton J.M., & Roberts B.J. (2016). Population Dynamics and Community Composition of Ammonia Oxidizers in Salt Marshes after the Deepwater Horizon Oil Spill. *Frontiers in Microbiology*, 7. 10.3389/fmicb.2016.00854 PMC4899434 27375576

[92] Turner R.E., & Bodker J.E. (2016). The effects of N, P and crude oil on the decomposition of Spartina alterniflora. *Wetlands Ecology and Management*, 24(3), 373–380.

[93] Rabalais N.N., & Turner R.E. (2016). Effects of the Deepwater Horizon Oil Spill on Coastal Marshes and Associated Organisms. *Oceanography*, 29(3), 150–159.

[94] Hester M.W., Willis J.M., Rouhani S., Steinhoff M.A., & Baker M.C. (2016). Impacts of the Deepwater Horizon oil spill on the salt marsh vegetation of Louisiana. *Environmental Pollution*, 216, 361–370. doi: 10.1016/j.envpol.2016.05.065 27299994 10.1016/j.envpol.2016.05.065

