# Supplementary figures and images for: Oiling accelerates loss of salt marshes, southeastern Louisiana
Source: PLoS One. 2017 Aug 2;12(8):e0181197. doi: 10.1371/journal.pone.0181197 (PMC5540489; doi:10.1371/journal.pone.0181197)

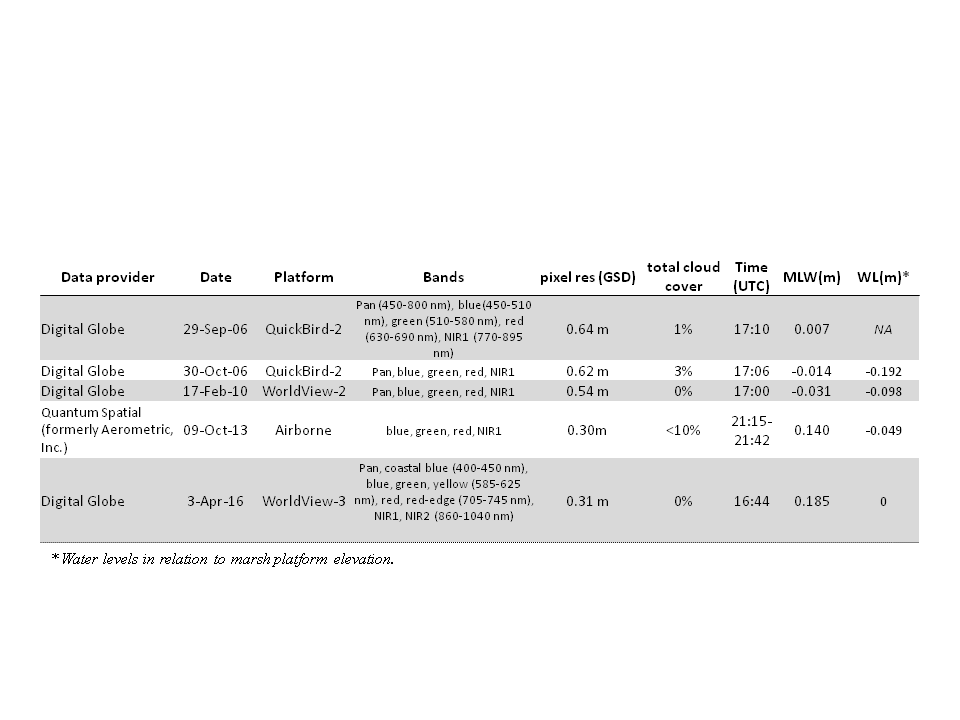

Supplement: S1 Table — (TIF) [file pone.0181197.s001.tif]

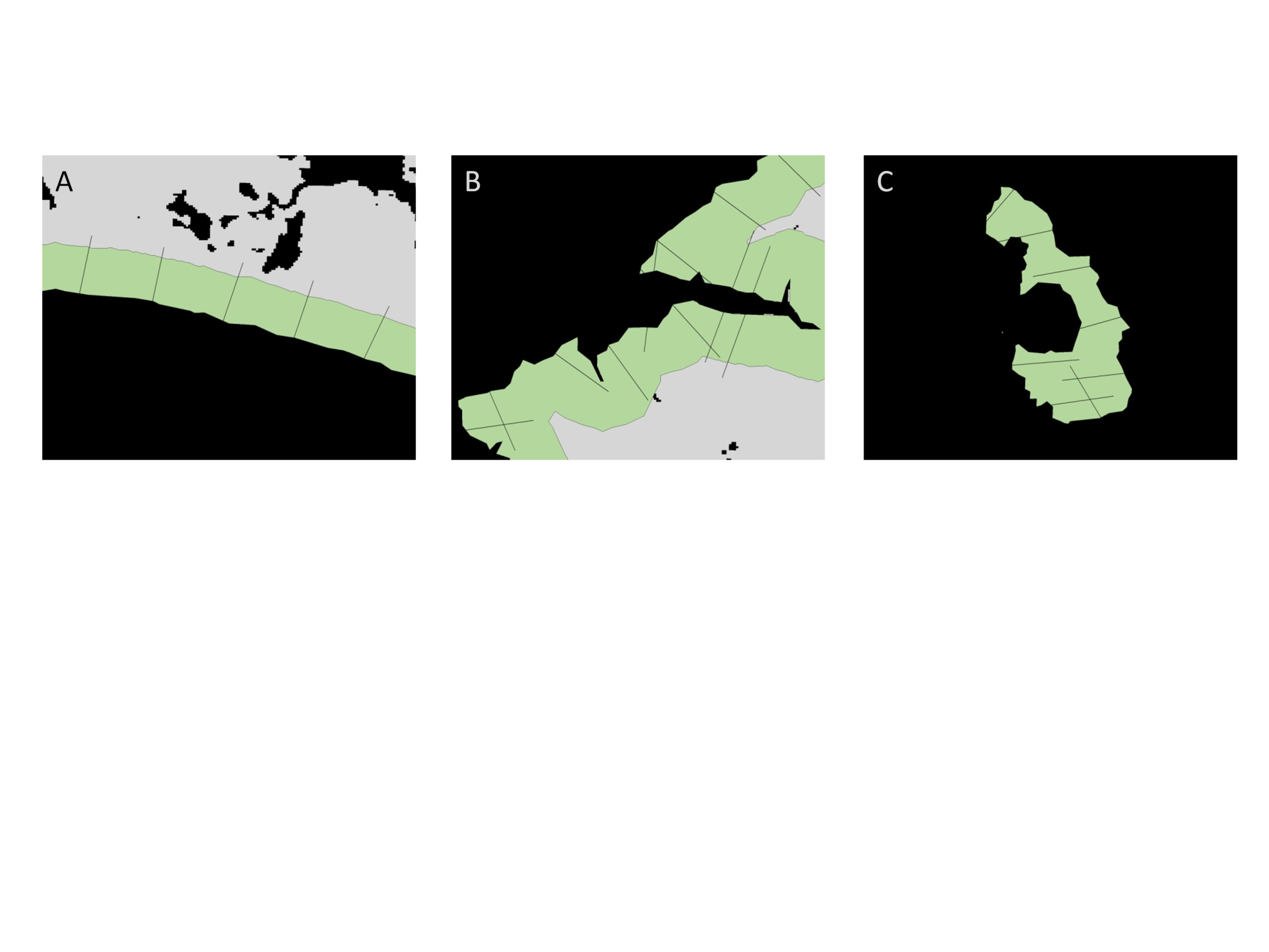

Supplement: S1 Fig — Examples of shoreline zones (green) and the onshore transects (black lines) that were used to segment the shoreline into reaches. For shorelines that were relatively linear (A), the transects segmented the shoreline into 100m reaches. However, for undulating shorelines (B), onshore transect direction was irregular and at times overlapping, creating shorter onshore reaches. Additionally, transect generation on small islands (C) commonly resulted in inconsistent reach lengths, and in some cases, required manual removal of selective transects. (TIF) [file pone.0181197.s003.tif]
